# Effect of fluoride application during radiotherapy on enamel demineralization

**DOI:** 10.1590/1678-7757-2018-0044

**Published:** 2018-12-10

**Authors:** Camila de Carvalho Almança Lopes, Carlos José Soares, Vitor Carvalho Lara, Victor Elias Arana-Chavez, Priscilla Barbosa Soares, Veridiana Resende Novais

**Affiliations:** 1 Universidade Federal de Uberlândia, Faculdade de Odontologia, Uberlândia, Minas Gerais, Brasil.; 2 Universidade Federal de Uberlândia, Faculdade de Odontologia, Departamento de Dentística e Materiais Odontológicos, Uberlândia, Minas Gerais, Brasil.; 3 Universidade Federal do Triângulo Mineiro, Faculdade de Medicina, Setor de Radioterapia, Uberaba, Minas Gerais, Brasil.; 4 Universidade de São Paulo, Faculdade de Odontologia, Departamento de Biomateriais e Biologia Oral, São Paulo, São Paulo, Brasil.; 5 Universidade Federal de Uberlândia, Faculdade de Odontologia, Departamento de Periodontia, Uberlândia, Minas Gerais, Brasil.

**Keywords:** Dental Enamel, Fluorides, Tooth Demineralization, Radiotherapy

## Abstract

Radiation-related caries are one the most undesired reactions manifested during or after head and neck radiotherapy. Fluoride application is an important strategy to reduce demineralization and enhance remineralizaton.

**Objective:**

To evaluate the effect of the topical application of fluoride during irradiation on dental enamel demineralization.

**Material and Methods:**

Thirty molars were randomly divided into three groups: Non-irradiated (NI), Irradiated (I), Irradiated with fluoride (IF). Each group was subdivided according to the presence or absence of pH-cycling (n=5). In the irradiated groups, the teeth received 70 Gy. The enamel's chemical composition was measured using Fourier Transform Infrared Spectrometry (organic matrix/mineral ratio - M/M and relative carbonate content - RCC). Vickers microhardness (VHN) and elastic modulus (E) were evaluated at three depths (surface, middle and deep enamel). Scanning electron microscopy (SEM) was used to assess the enamel's morphology.

**Results:**

The FTIR analysis (M/M and RCC) showed significant differences for irradiation, pH-cycling and the interaction between factors (p<0.001). Without pH-cycling, IF had the lowest organic matrix/mineral ratio and relative carbonate content. With pH-cycling, the organic matrix/mineral ratio increased and the relative carbonate content decreased, except for IF. VHN was influenced only by pH-cycling (p<0.001), which generated higher VHN values. ANOVA detected significant differences in E for irradiation (p<0.001), pH-cycling (p<0.001) and for the interaction between irradiation and pH-cycling (p<0.001). Increased E was found for group I without pH-cycling. With pH-cycling, groups I and IF were similar, and showed higher values than NI. The SEM images showed no morphological changes without pH-cycling. With pH-cycling, fluoride helped to maintain the outer enamel's morphology.

**Conclusions:**

Fluoride reduced mineral loss and maintained the outer morphology of irradiated and cycled enamel. However, it was not as effective in preserving the mechanical properties of enamel. Radiotherapy altered the enamel's elastic modulus and its chemical composition.

## Introduction

Of the several therapeutic modalities, radiotherapy is one of the most widely used in the treatment of head and neck cancer ^1^ . Patients undergoing radiotherapy often develop early and late oral radiation-induced complications such as mucositis, hyposalivation and subsequent xerostomia, taste loss, osteoradionecrosis and radiation-related caries. ^1^ Radiation-related caries are a lifelong risk, and not only during or shortly after the treatment. ^2^ Dental caries have a rapid onset and progression, and may be accompanied by enamel delamination, leading to crown amputation. ^2^ ^-^ ^4^ The occurrence and severity of radiation caries are related to the changes in the quality and quantity of saliva, changes in the oral microbiota, difficulty in promoting oral hygiene, and increasingly cariogenic diet. ^3^ ^-^ ^5^ In synergy, there are direct effects on dental tissue, including changes in the crystalline structure, dentinoenamel junction (DEJ), acid solubility of the enamel, enamel and dentin microhardness, and tensile strength. ^6^ ^,^ ^7^

An important strategy to prevent dental caries is to reduce demineralization and enhance remineralization. ^8^ ^,^ ^9^ There are several fluoride-containing products on the market for professional application and their anticariogenic effect will depend on the product formed in the enamel and its retention on the surface of the enamel over time. ^8^ There is no universal protocol for the treatment of radiation-related caries, however, the importance of fluoride is well recognized. ^10^ The daily topical application of 1% neutral sodium fluoride gel with custom-made fluoride carriers has been shown to reduce post radiation-related caries. ^11^ However, no study has evaluated the effect of the neutral fluoride gel in contact with the enamel during the ionizing radiation process on the enamel's properties.

Therefore, the aim of this *in vitro* study was to evaluate the effect of the topical application of fluoride during irradiation on the chemical composition (ATRFTIR), mechanical properties (Vickers Microhardness - VHN and Elastic Modulus - E) and morphology (SEM) of sound and pH-cycled enamel. The null hypothesis tested was that the fluoride applied during irradiation would have no effect on the enamel's properties.

## Material and methods

After approval from the Research Ethics Committee (No. 37868814.6.0000.5152), freshly extracted human third molars from 18-25 year-old individuals were collected and stored in refrigerated deionized water (4°C), up to 3 months after extraction. Before the experimental procedures, the teeth were cleaned and examined under a stereoscopic microscope (Leica MS5, Leica Microscopy Systems Ltd; Heerbrugg, Switzerland) to show evidence of caries, enamel hypoplasia and other defects. Thirty sound teeth were selected and randomly divided into three groups (n=10): NI - non-irradiated, I - irradiated in relative humidity, IF - irradiated with fluoride. Within each group, the teeth were subdivided into 2 subgroups (n=5) according to the presence of pH-cycling: with and without pH-cycling. The use of relative humidity was an attempt to simulate the oral condition of hyposalivation which patients with head and neck cancer undergo within the first week of radiotherapy and that persists throughout treatment. ^12^

The teeth of the irradiated groups received 70 Gy from tridimensional conformal radiotherapy by using a Linear Accelerator (Clinac 600C, Varian Medical Systems; Corona, CA, USA), fractioned into 2 Gy/day, 5 days a week, for 7 weeks. Relative humidity was achieved by placing a wet compress over the teeth's crowns during irradiation. For group IF, 1% manipulated neutral fluoride gel (Biopharma; Uberlândia, MG, Brazil) was applied on the enamel 4 minutes before the radiation procedure, and was maintained throughout the entire irradiation process, totalizing 5 minutes of contact. ^13^ After this, the specimens were rinsed to remove the fluoride gel, and subsequently stored in deionized water that was changed weekly.

### Specimens' preparation

**Figure 1:**
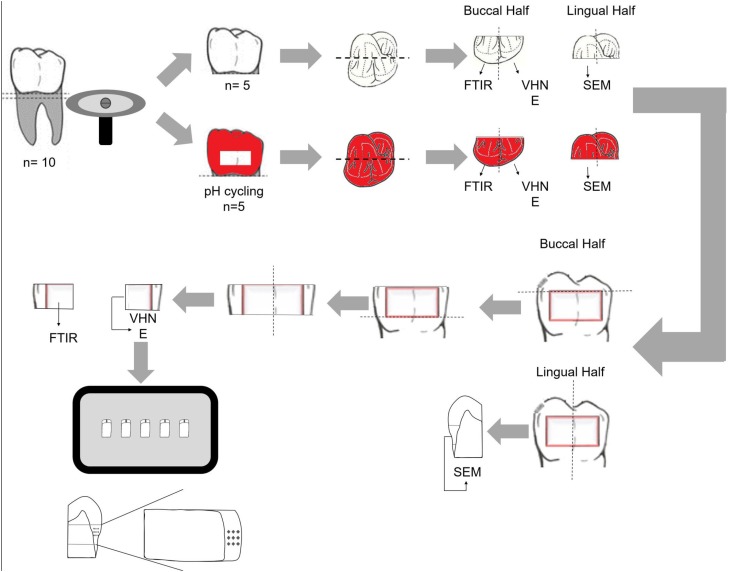
Schematic illustration of the experimental design. The teeth were sectioned for analyses using Fourier Transform Infrared Spectroscopy (FTIR) (n=5); Vickers microhardness and elastic modulus (n=5); and for morphological analyses (SEM) (n=3)

The roots of all teeth were removed 1 mm below the cementoenamel junction, by using a water-cooled diamond saw (Isomet, series 15HC diamond, Buehler Ltd.; Lake Bluff, IL, USA) and a precision saw (Isomet 1000, Buehler; Lake Bluff, IL, USA). On five teeth in each group that was submitted to pH-cycling, an area measuring 9×3 mm was demarcated on the buccal and lingual enamel surfaces, approximately 1.0 mm above the cementoenamel junction. Two layers of an acid-resistant varnish (Colorama Maybelline; São Paulo, SP, Brazil) were applied outside the demarcated area. Each crown was sectioned in the mesial/distal direction, resulting in two halves: buccal and lingual. All buccal halves were cut into two slabs (3×2.7 mm) obtained from the middle third ( Figure 1 ), designed for FTIR and dynamic microindentation tests. The lingual half was sectioned and only the mesial quarter was used for the SEM analysis.

### pH-cycling

After the irradiation procedure, the specimens designated for pH-cycling were subjected to dynamic demineralization and to the remineralization cycling model as proposed previously. ^14^ This pH-cycling challenge consisted of periods of immersion in a demineralizing solution (6 hours) and then in a remineralizing solution (18 hours), at 37°C. The demineralization solution was composed of 2.0 mmol/L of CaCl_2_, 2.0 mmol/L of NaH_2_PO_4_, and 75 mmol/L of acetic acid with 4.3 pH, adjusted with KOH (1 M) *per* liter of solution, and the remineralization solution was composed of 1.5 mmol/L of CaCl_2_, 0.9 mmol/L of NaH_2_PO_4_, 150 mmol/L of KCl with 7.0 pH, adjusted with KOH (1 M) per liter of solution. The specimens were rinsed in deionized water between solution exchanges. This procedure was carried out for 14 days. At the end of each 5 consecutive days of cycling, the specimens were kept in a remineralizing solution for 2 days. The solutions were renewed on the fifth day.

For the assessment of the measured parameters, the examiner was blinded to the groups submitted to radiotherapy.

### Fourier Transform Infrared Spectroscopy (FTIR)

The enamel's chemical composition was verified using Fourier Transform Infrared Spectroscopy (FTIR Vertex 70 - Bruker; Ettlingen, Baden-Württemberg, Germany) equipped with an accessory that allowed spectrum acquisitions in the Attenuated Reflectance (ATR) mode (n = 5). The spectra were recorded in the 400-4,000 cm^−1^ range at a 4 cm^−1^ resolution. The testing surface was positioned against the diamond crystal of the FTIR unit and pressed with a force gauge at constant pressure to facilitate contact. The sample was scanned 128 times in each FTIR measurement, and the spectrum acquired was the average of all scans. The spectra were recorded and analyzed with the OPUS 6.5 software (Bruker; Ettlingen, Baden-Württemberg, Germany). The background was subtracted, and after baseline correction using rubber band correction and normalization, the area under each band was integrated by using the appropriate tools of the program. Each band was normalized by the phosphate band (1,190-702 cm^−1^). The FTIR spectra were further analyzed by calculating the following parameters: organic matrix/mineral ratio, relative carbonate content. ^15^ The organic matrix/mineral ratio, expressed by the ratio between integrated areas of protein amide I – 1653 cm^−1^ and phosphate v_1_, v_3_ stretching mode – 960cm^−1^ and 1040 cm^−1^, was used to evaluate the amount of organic matrix with respect to inorganic matrix. The relative carbonate content, expressed by the ratio of the integrated areas of the two strongest carbonate peaks at 1460 and 1425 cm^−1^ and phosphate v_1_, v_3_, indicated the extent of carbonate incorporated into the hydroxyapatite of a particular specimen.

### Dynamic microindentation test

The specimens' preparation and the experimental protocol were performed as previously described by Soares, et al. ^16^ (2014). The specimens (n = 5) were embedded in polyester resin (Instrumental Instrumentos de Medição Ltda; São Paulo, SP, Brazil). The specimen was positioned on a glass plate with the cutting surface downward, and then fixed using an adhesive system (Single Bond 2, 3M-Espe; St. Paul, MN, USA). A thin layer of the adhesive system was applied to the glass surface and the specimen was stabilized using digital pressure, followed by light activation for 20 s (Demetron 501, Kerr; Orange, CA, USA). After this, a metal tube (Metalon Indústrias Reunidas; Nova Iguaçu, RJ, Brazil), 50 mm long, 30 mm wide and 10 mm tall, was fixed around the specimens (five specimens *per* block) with wax (Wilson; Cotia, SP, Brazil). After inserting and curing the polyester resin, the specimens' surfaces were sanded using silicon-carbide abrasive papers (#600, 800, 1200, 1500 and 2000 grit; Norton; Campinas, SP, Brazil) and polished with metallographic diamond pastes (6, 3, 1, and *¼* μm grit, Arotec; São Paulo, SP, Brazil). The specimens were washed with deionized water and cleaned ultrasonically in absolute alcohol for 5 minutes between each metallographic diamond paste polishing procedure.

Vickers Microhardness (VHN) and the Elastic modulus (E) of the enamel were assessed using a dynamic microhardness indenter (CSM Micro-Hardness Tester, CSM Instruments; Peseux, Switzerland) equipped with a Vickers diamond with pyramidal geometry and quadrangular base. Three indentations were performed in three different regions of the enamel: 40 μm from its outer edge (superficial enamel), at one-half the thickness of the enamel (middle enamel), and 40 μm from the dentinoenamel junction (deep enamel) for each sample. ^17^ The indentation was carried out with controlled force, and at a constant speed ranging between 0 and 500 mN in 60 s intervals. Maximum force (500 mN) was held for 15 s. Then, the force was gradually removed from 500 mN to 0 mN in 60 s intervals. The VHN and E were calculated by the software provided with the indentation apparatus and the mean value of the indentations for each depth was recorded.

### Scanning Electron Microscopy (SEM)

A longitudinal fracture was made on the enamel's surface in lingual hemi-sections. The specimens were fixed in glutaraldehyde solution in cacodylate buffer for 2 hours, dehydrated in increasing concentrations of ethanol (30%, 50%, 75%, 80%, 90%, 95%, and 100%), immersed in hexamethyldisilazane (HMDS) for 10 minutes and kept in an incubator for 24 hours ^18^ . Subsequently, the specimens were fixed on stubs with the cutting side upwards using double-sided adhesive carbon tape (Electron Microscopy Sciences; Washington, PA, USA) and sputter-coated with gold in a vacuum metallizing machine (Bal-Tec SCD-050, Leica Microsystems; Wetzlar, Germany). The specimens were examined with a scanning electron microscope (LEO 450 model, LEO Electron Microscopy Ltd.; Cambridge, United Kingdom), operated at 15 kV using the secondary electron detector.

### Statistical analysis

The organic matrix/mineral ratio, relative carbonate content, VHN and E values were tested for normal distribution (Shapiro-Wilk, α<0.05) and equality of variances (Levene test, α<0.05), followed by parametric statistical tests. The organic matrix/mineral ratio and the relative carbonate content were analyzed using two-way ANOVA. Three-way analysis of variance (ANOVA) was performed for the VHN and E values and multiple comparisons were made using Tukey test. For all tests, a 0.05 level of statistical significance was used, and all statistical analyses were carried out using a statistical package (SigmaPlot^®^ System, version 12.0, Systat Institute Inc.; San Jose, CA, USA).

## Results

### FTIR analysis

**Figure 2:**
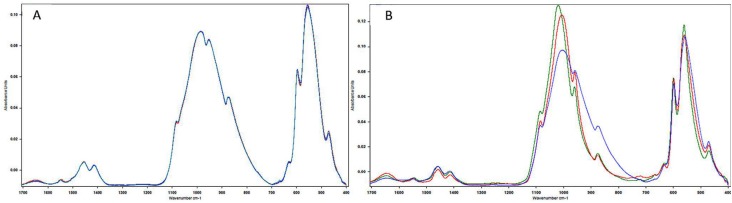
Absorbance spectra for the non-irradiated (green line), irradiated (red line) and fluoride-irradiated (blue line) enamel, without pH-cycling (A) and after pH-cycling (B). Amide I (1653 cm^−1^), Carbonate v3 (1460 and 1425 cm^−1^), Phosphate v1 and v3 (960 and 1040cm^−1^)

The spectra for groups NI, I and IF with and without pH-cycling are shown in Figure 2 a and b respectively. Maintenance of the main bands characteristic of enamel was observed. Without pH-cycling, the spectra almost overlapped, except for the amide I band. The irradiated enamel showed the greatest decrease in the amide I intensity band. After pH-cycling, carbonate loss was observed, however, it was less pronounced in the irradiated enamel that received fluoride.

The mean values of the integrated area of each chemical component analyzed are presented in Table 1 . The mean and standard deviation values of the organic matrix/mineral ratio and of the relative carbonate content according to irradiation and pH-cycling are shown in Table 2 . For the organic matrix/mineral ratio and relative carbonate content, 2-way ANOVA showed statistical significance for irradiation, pH-cycling and the interaction between the factors (p<0.001). Without pH-cycling, higher organic matrix/mineral ratio and relative carbonate content values were found for group I when compared with IF, while group NI was similar to both groups. The organic matrix/mineral ratio increased and the relative carbonate content decreased with pH-cycling for groups NI and I, and remained constant for group IF. After pH-cycling, groups NI and I showed the highest organic matrix/mineral ratio values and the lowest relative carbonate content values. Group IF showed the lowest organic matrix/mineral ratio and the highest relative carbonate content values.

**Table 1:** Mean values of the integrated area of each chemical component analyzed using Fourier Transform Infrared Spectroscopy (FTIR)

Irradiation	Amide I		PO v1 e v3		CO v3	
	No pH cycling	pH cycling	No pH cycling	pH cycling	No pH cycling	pH cycling
Non-irradiated	0.148	0.359	8.630	13.237	1.307	0.912
Irradiated	0.167	0.346	8.788	13.197	1.356	0.743
Irradiated in fluoride	0.116	0.116	8.829	10.628	1.314	1.034

**Table 2:** Means and standard deviations for M/M, RCC of the enamel comparing type of irradiation and pH-cycling

Irradiation	Organic matrix/mineral ratio		Relative Carbon Content	
	No pH cycling	pH cycling	No pH cycling	pH cycling
Non-irradiated	0.017±0.004^Bab^	0.027±0.001^Aa^	0.153±0.025^Aab^	0.069±0.009^Bb^
Irradiated	0.020±0.002^Ba^	0.026±0.003^Aa^	0.154±0.016^Aa^	0.056±0.006^Bc^
Irradiated in fluoride	0.013±0.004^Ab^	0.011±0.002^Ab^	0.149±0.007^Ab^	0.150±0.016^Aa^

* Different uppercase letters show significant differences in horizontal. Different lowercase letters show significant differences in vertical

### Dynamic indentation test

The means and standard deviations for the VHN values according to the type of irradiation, pH-cycling and enamel depths are shown in Table 3 . Three-way ANOVA revealed statistical significance for pH-cycling (p<0.001). However, no significance was found for the irradiation factor (p=0.429), enamel depth (p=0.058), or for the interactions between irradiation and pH- cycling (p = 0.108), irradiation and enamel depths (p=0.408), pH-cycling and enamel depths (p=0.718) and between irradiation, pH-cycling and enamel depths (p=0.932). Lower VHN values were found for all groups without pH-cycling.

**Table 3:** Means and standard deviations (±) for VHN (N/mm^2^) values of the enamel comparing type of irradiation, pH-cycling and enamel depths

Irradiation	Enamel depth	No pH cycling		pH cycling	
		Mean (±)	Pooled Average	Mean (±)	Pooled Average
Non-irradiated	Superficial enamel	486.7±133.1	453.0±112.9^B^	619.7±139.5	625.1±151.9^A^
	Middle enamel	466.2±103.3		613.6±122.1	
	Deep enamel	406.1±102.5		642.0±194.0	
Irradiated	Superficial enamel	608.1±73.0	525.0±71.6^B^	676.2±68.2	589.7±59.9^A^
	Middle enamel	512.1±79.3		561.4±60.2	
	Deep enamel	454.8±62.6		531.5±51.3	
Irradiated in fluoride	Superficial enamel	489.3±37.8	479.3±43.1^B^	606.9±202.8	582.3±192.7^A^
	Middle enamel	469.1±28.1		563.1±178.2	
	Deep enamel	479.6±63.3		576.9±197.1	

* Different uppercase letters indicate significant difference between the pH cycling

The means and standard deviations for the E values according to the type of irradiation, pH-cycling, and enamel depths are shown in Table 4 . Three-way ANOVA revealed significant effect for the irradiation factor (p<0.001), pH-cycling (p<0.001) and for the interaction between irradiation and pH-cycling (p<0.001). However, no significant difference was found for enamel depths (p=0.293) and for the interactions between irradiation and enamel depths (p=0.850), pH-cycling and enamel depths (p=0.619), and between irradiation factor, pH-cycling and enamel depths (p = 0.721). When comparing the groups without pH-cycling, group I had the highest E values, followed by groups IF and NI. When comparing the groups with pH-cycling, the E values of groups I and IF were similar and higher than those of group NI. The E values for group I remained constant with or without pH-cycling.

**Table 4:** Means and standard deviations (±) for E (GPa) values of the enamel comparing type of irradiation, pH-cycling and enamel depths

Irradiation	Enamel depth	No pH cycling		pH cycling	
		Mean (±)	Pooled Average	Mean (±)	Pooled Average
Non-irradiated	Superficial enamel	45.5±3.5	44.3±3.3^Bc^	59.5±1.4	61.3±2.9^Ab^
	Middle enamel	45.1±3.3		61.5±5.0	
	Deep enamel	42.4±3.1		63.0±2.4	
Irradiated	Superficial enamel	70.4±3.8	68.6±2.3^Aa^	73.2±7.4	69.9±7.1^Aa^
	Middle enamel	68.5±2.2		69.1±7.8	
	Deep enamel	66.9±0.9		67.5±6.1	
Irradiated in fluoride	Superficial enamel	62.9±3.7	61.6±2.9^Bb^	75.2±11.9	74.1±11.3^Aa^
	Middle enamel	61.2±4.0		73.9±12.4	
	Deep enamel	60.8±4.4		73.1±9.6	

* Different uppercase letters show significant differences between pH cycling. Different lowercase letters show significant differences for irradiation factor

### SEM analysis

**Figure 3:**
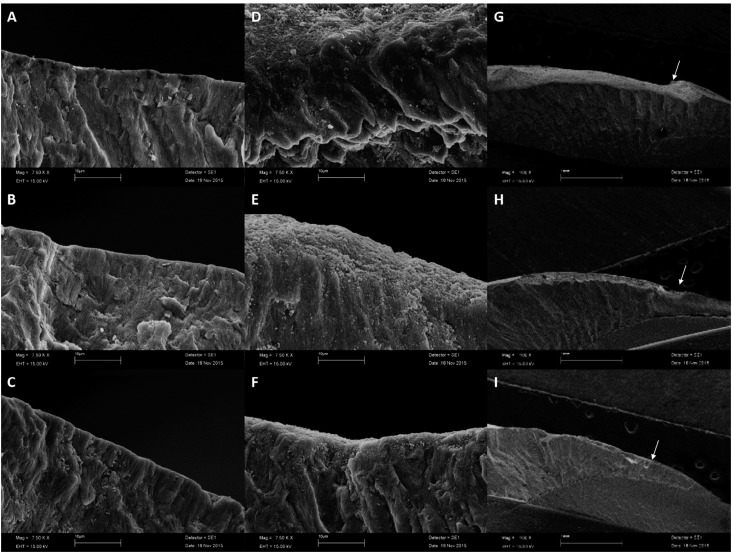
Electron micrographs of the enamel. The images were obtained via scanning electron microscopy at 7.500x (A, B, C, D, E, F) and 100x (G, H, I) magnifications. A, B. C - Images of the surface enamel without pH-cycling; A - Non-irradiated enamel; B - Enamel irradiated in humidity; C - Enamel irradiated in fluoride. D, E, F, G, H, I - Images of the surface enamel after pH-cycling; D and G - Non-irradiated enamel; E and H - Enamel irradiated in humidity; F and I - Enamel irradiated in fluoride. The arrows indicate the loss of discontinuity of the outer morphology

The enamel of all groups featured well-organized prisms, surrounded by interprismatic regions ( Figure 3 A-C). The enamel exposed to pH-cycling resulted in discontinuity of the buccal contour that was more accentuated in group NI than in group I. No evident discontinuity was observed in the enamel irradiated with fluoride ( Figure 3 D-I).

## Discussion

The null hypothesis tested in this study was rejected. The radiation from the linear accelerator altered the chemical composition and elastic modulus of the enamel, and pH-cycling altered its chemical composition and mechanical properties, whereas fluoride attenuated demineralization. Depending on the localization of the malignancy, the salivary glands, oral mucosa and jaws inevitably have to be included in the radiotherapy field ^1^ ^,^ ^2^ , causing damage to oral function, and interfering in the patient's quality of life. ^5^ Radiation-related caries are an atypical pattern of dental caries due to the combination of both hyposalivation and the direct effects on the hard dental tissue. ^3^ ^-^ ^5^ Under some conditions, the teeth may be completely lost within short periods ^2^ . There is still no consensus about how the dental hard tissues changed and how the changes may contribute to rapid deterioration of the teeth. ^5^ ^,^ ^19^

The literature shows several variables in the form of simulating *in vitro* radiotherapy. Third molars from 1825 year-old patients were used to control variability, since it is known that patient age can interfere in the elastic modulus and hardness of the enamel. ^20^ It has been previously reported that physiological saline solution, artificial saliva or distilled water were used as storage solution. ^12^ ^,^ ^19^ In this study, deionized water was used in attempt to simulate the oral conditions of a patient whose head and neck were irradiated soon after the start of irradiation ^21^ , where there is a decrease in saliva flow and consequently there is lower availability of calcium and phosphate in the medium for enamel remineralization.

The mechanical properties of the enamel were assessed using a dynamic indentation test. Radiotherapy increased the VHN values, in spite of not being statistically significant, and the E values. An important aspect of radiotherapy is radiolysis, wherein radiation interacts with water. Despite enamel being mostly composed of mineral phase, it still has 4-6% of organic constituents, such as protein, peptides and water. ^22^ When radiolysis occurs, reactive unstable free radicals H^+^ and OH^-^ are released and then it can interact with other ions to produce new compounds. ^23^ This might explain the increase in amide I, phosphate and carbonate content after exposure to radiation, contributing to the altered mechanical properties. Moreover, the degradation of the water may have led to a dehydrated and more hypermineralized enamel tissue, making it susceptible to the formation of cracks ^24^ . All these changes made the enamel more friable and could be the mechanism responsible for its delamination, frequently observed after radiotherapy. ^25^ One of the studied factors was enamel depth. Although there were no statistical differences, group IF showed certain constancy in the VHN and *E* values at the three depths evaluated, which did not occur in groups NI and I, independently of pH-cycling. Once again, this demonstrated the importance of fluoride in the maintenance of the enamel's properties, ensuring its anti-caries effect. ^26^

Both irradiation and pH-cycling had a significant effect on the organic matrix/mineral ratio and on the relative carbonate content. Group IF without pH-cycling had the lowest values of both parameters analyzed, exhibiting a more mineralized tissue than the other groups. This may be explained by the capacity of fluoride ions to substitute hydroxyl ions in the hydroxyapatite, reducing the space filled by the organic matrix. ^25^ On the other hand, radiotherapy promoted an increase in carbonate content, causing crystalline deformation and a tissue that was more soluble in acid. This slight increase in the enamel's acid solubility was also reported in the literature. ^27^ After the pH challenge, groups NI and I showed the highest organic matrix/mineral ratio values, since the acid challenge led to demineralization, exposing a larger portion of the organic matrix. Group IF maintained the organic matrix/mineral ratio and the relative carbonate content values at levels similar to those before and after pH-cycling, showing that fluoride protected the enamel against acid by diminishing its solubility. ^28^ In the same way, previous studies have noted the importance of fluoride in the protection of these high-risk patients from caries in combination with good oral hygiene. ^7^ ^,^ ^11^ ^,^ ^29^ ^,^ ^31^ Kielbassa, et al. ^7^ (1997) also stated that neutral or nearly neutral fluoride-containing gels or solutions should preferably be used.

The pH-cycling models were developed to simulate the events associated with the caries' onset process, such as dynamic variations in mineral saturation and pH. ^32^ With these models it is possible to isolate individual factors under controlled conditions, which would be extremely challenging to do under *in vivo* conditions. ^33^ Nevertheless, this type of model has its limitations, for example, it does not employ saliva and biofilm. However, it is still widely used due to its simplicity and because the method produces lesions which are histologically similar to those naturally developed in the enamel, also being used to assess fluoride's mode of action. ^34^

In the present study, a pH-cycling model was chosen to simulate the cariogenic challenge undergone by irradiated patients affected by head and neck cancer. Calcium-deficient regions rich in carbonates are especially susceptible to acid attacks by hydrogen ions during demineralization, ^35^ in other words, during demineralization carbonate is lost. On the other hand, during remineralization, carbonate is removed ^35^ and salivary phosphate ions are incorporated into the enamel. ^9^ In this study, FTIR showed a decrease in the content of carbonate and increase in that of phosphate, and consequently a decrease in the relative carbonate content, ratifying the increased VHN and E values. ^36^ Thus, enamel remineralization was established, possibly due to the two days when the specimens remained in a remineralizing solution after the first five days of cycling. Argenta, Tabchoury and Cury ^32^ (2003) advocated keeping the specimens in the remineralizing solution for this period, since it preserved the enamel's surface layer allowing the VHN to be determined. The phosphate incorporated during remineralization is substantially more soluble in acid than dental enamel and hydroxyapatite. ^37^ Thus, the NI and I groups that had higher increase in phosphate content during pH-cycling will have greater dissolution of its mineral content in the event of a sharp drop in pH. On the other hand, fluoride was incapable of protecting the enamel against the increase in the elastic modulus when pH-cycling was present, thus resembling the irradiated enamel.

This finding is opposite to those of previous studies, which have shown no differences between irradiated and nonirradiated enamel lesions ^2^ ^,^ ^3^ probably because these authors used *in situ* experiments, with participants in good general health with normal salivary flow. There are some differences in the clinical oral environment of irradiated and non-irradiated patients, especially with regard to salivary flow. It should be considered that remineralization occurs only with resting salivary flow. ^3^ Thus, in patients with irreversible damages to the salivary glands or low resting flow rate, remineralization is impaired, which appears to promote an increase in the incidence of radiation-related caries.

No micromorphological alterations in the enamel were revealed after irradiation. However, when the tooth was subjected to pH-cycling, the surfaces evaluated showed aspects of demineralization, seen as discontinuity of the outer morphology of the enamel ( Figure 3 G-H). The enamel irradiated with fluoride showed the lowest degree of demineralization ( Figure 3 G-H). Since fluoride helps to decrease the solubility of enamel when exposed to acid challenges, ^36^ this process was expected to occur in a less pronounced way than in enamel that was not exposed to fluoride.

Clinical studies are needed to confirm the results of the *in vitro* studies and to create protocols with the purpose of minimizing or counteracting radiation-related caries and damages to the dental hard tissue. Nevertheless, the use of fluoride during radiation therapy is an applicable approach for patients with head and neck cancer, and a possible strategy for this procedure is the use of fluoride in trays.

## Conclusions

The findings of this *in vitro* study demonstrated that the application of 1% neutral fluoride gel during the irradiation procedure maintained the morphological and chemical integrity of the irradiated and pH-cycled enamel. However, topical fluoride was not as effective in preserving its mechanical properties.
